# Light Limitation within Southern New Zealand Kelp Forest Communities

**DOI:** 10.1371/journal.pone.0123676

**Published:** 2015-04-22

**Authors:** Matthew J. Desmond, Daniel W. Pritchard, Christopher D. Hepburn

**Affiliations:** Department of Marine Science, University of Otago, Dunedin, New Zealand; University of Waikato, NEW ZEALAND

## Abstract

Light is the fundamental driver of primary productivity in the marine environment. Reduced light availability has the potential to alter the distribution, community composition, and productivity of key benthic primary producers, potentially reducing habitat and energy provision to coastal food webs. We compared the underwater light environment of macroalgal dominated shallow subtidal rocky reef habitats on a coastline modified by human activities with a coastline of forested catchments. Key metrics describing the availability of photosynthetically active radiation (PAR) were determined over 295 days and were related to macroalgal depth distribution, community composition, and standing biomass patterns, which were recorded seasonally. Light attenuation was more than twice as high in shallow subtidal zones along the modified coast. Macroalgal biomass was 2–5 times greater within forested sites, and even in shallow water (2m) a significant difference in biomass was observed. Long-term light dose provided the best explanation for differences in observed biomass between modified and forested coasts, with light availability over the study period differing by 60 and 90 mol photons m^−2^ at 2 and 10 metres, respectively. Higher biomass on the forested coast was driven by the presence of larger individuals rather than species diversity or density. This study suggests that commonly used metrics such as species diversity and density are not as sensitive as direct measures of biomass when detecting the effects of light limitation within macroalgal communities.

## Introduction

The availability of photosynthetically active radiation (PAR), henceforth referred to as light, in the world’s coastal seas is highly variable and in many cases substantially modified by anthropogenic activities [1,2]. The discharge of wastewater, dumping of dredge spoil and the clearance of land for agricultural, horticultural and urban purposes all increase sediment loading, and as a result turbidity, in coastal seas [2–5]. Light availability acts in concert with nutrients [6], temperature [7], herbivory [8] and wave exposure [9] to drive photosynthetic carbon fixation and ultimately, coastal primary production [10–12]. Changes to the light environment of coastal seas could have major implications for the productivity of coastal food webs which support an estimated 90% of the world’s fisheries [13] and therefore, light availability deserves closer investigation [10,14,15].

Macroalgae are major primary producers in coastal seas, in some cases they provide up to 90% of total carbon to coastal food-webs [16–18]. Macroalgae are also considered foundation species or ecosystem engineers as they form complex three dimensional habitat [7,19]. This habitat supports an array of organisms through a range of life history stages and provides numerous ecosystem services [7,20,21]. Assuming the presence of suitable substrate and lack of significant grazing pressure, light penetration into the water column ultimately controls macroalgal depth distribution, and as a result potential primary productivity and community structure [17,22]. Productivity and overall ecosystem functioning of macroalgal communities is therefore greatly affected by changes in the underwater light environment [3,23–28]. Globally macroalgal distribution has been estimated to be limited by light in 34–58% of the non-polar coastal regions [17]. With predicted increases in coastal anthropogenic activity this estimate is likely to increase [1,29].

Macroalgae employ a range of mechanisms to deal with variability in the quality and quantity of light reaching the benthos [3,28,30,31]. Responses of macroalgae to such variability can occur as regulation, e.g. dynamic photoinhibition in periods of high light stress [32,33], acclimation, e.g. varying concentrations of photosynthetic pigments [34–36], and / or adaption, e.g. changes to thallus morphology which alter the efficiency of light absorption per unit of photosynthetic tissue [32,37]. However, changes in turbidity, especially those caused by anthropogenic disturbance, can occur over relatively short time scales and in some instances species may not have the capacity to respond [38]. Light limitation has the potential to compress depth distributions of species [26,37,39], reduce growth rates [25,28] and decrease community complexity [40]. All of these changes culminate in a reduction of primary productivity and habitat availability as well as decreased ecosystem resilience to stress [41,42].

**Fig 1:**
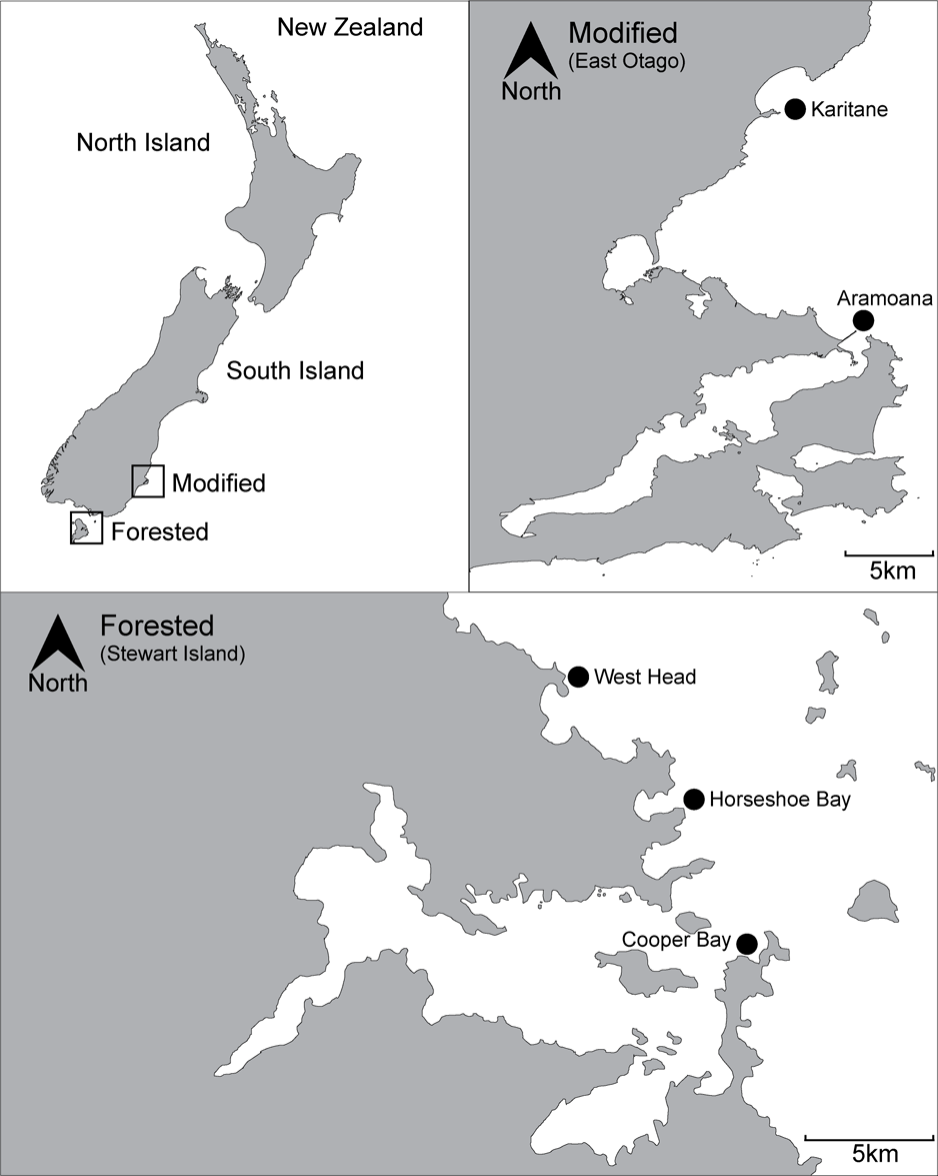
Location of study sites. Top left, New Zealand with the modified (East Otago) and forested (Stewart Island) coastlines highlighted. Top right, modified with Karitāne and Aramoana sites marked by black dot. Bottom, forested with West Head, Horseshoe Bay and Cooper Bay sites marked by black dot.

There is consensus that in-depth, long term investigations are critical to better understand how the underwater light environment influences the productivity and structure of such important coastal marine ecosystems [3,10,14,15,40,43] and there is growing concern regarding the loss of macroalgal dominated habitats worldwide [40,44]. The focus of this study was to quantify light in two shallow coastal reef ecosystems with varying underwater light regimes. This was achieved by comparing subtidal reef environments in southern New Zealand. One is associated with catchments containing intact mixed native podocarp forest (termed forested) on Stewart Island (Rakiura) and the other is a coast dominated by agriculture, forestry and urban development (termed modified) in East Otago (Fig 1). Mixed podocarp forests on Stewart Island are similar to those that once covered many coastal areas within New Zealand, in particular the southeast of the South Island which includes the East Otago region [45–47]. Data gathered were used to address the question: Does the availability of light explain coastal patterns in benthic macroalgal depth distribution, community composition and standing biomass? We hypothesised that sites associated with the forested catchment will receive a higher annual light dose and support greater macroalgal biomass, a more complex community structure and deeper depth limits for macroalgal species compared to sites associated with the modified catchment. This information is important in understanding processes controlling coastal primary productivity and can potentially be applied to support coastal and marine management initiatives.

## Materials and Methods

### Study Area

Field studies did not require any permit or permission and did not involve any protected or endangered species. This study employed a nested design by establishing five sites, two located along the East Otago coast of New Zealand’s South Island (low light, modified catchment) and three along the northern coast of Stewart Island (high light, forested catchment) (Fig 1). A limitation of such a design is that without spatially interspersed low light and highlight sites, which do not exist along this coastline and are extremely rare worldwide, biogeographical factors other than light may influence the trends observed in this study. Measures were taken to quantify potential confounding factors such as temperature, nutrient availability and grazing pressure, following this the chosen sites were shown to be highly comparable with the exception of their underwater light environment. Each site had a similar north to northeast aspect, was subject to similar levels of wave exposure and had a substrate of boulders, bedrock or a combination of both. The reef at each site had a similar moderate gradient sloping down to an approximate depth of 10–12 metres before reaching sand. Each reef system was inhabited by a variety of sub-canopy macroalgal species with *Macrocystis pyrifera* forming the canopy. The surrounding catchment areas of each site differed, Karitāne, (45° 38’ S, 170° 40’ E) (modified) was predominantly agricultural farmland and small patches of exotic forest, Aramoana (45° 46’ S, 170° 43’ E) (modified) was predominantly urban settlement and small scale agriculture, while the three sites of West Head (46° 50’ S, 168° 05’ E), Horseshoe Bay (46° 52’ S, 168° 08’ E) and Cooper Bay (46° 55’ S, 168° 10’ E) (forested) were native forest (Fig 1).

Total rainfall for the period of the study was 707mm within the modified coast and 1347mm within the forested coast (CliFlo 2014, NIWA). Nutrient availability during the period of this study was relatively similar between coasts with the modified coast having 1.15 and 11 μmol/L of nitrogen during summer and winter, respectively, while the forested coast had 1.75 and 9 μmol/L for the same periods, these results are similar to those seen in past studies in the same area [48,49]. Mean water temperature between modified (295 days, two sites, *n* = 590) and forested (295 days, three sites, *n* = 885) coasts was similar, 13.0 and 12.8°C at two metres depth and 12.7 and 12.8°C at 10 metres depth, respectively. Sites were sheltered from the prevailing southwest swell and the presence of *Durvillaea spp*. and *Macrocystis pyrifera* indicated a moderate level of wave exposure [49,50]. Preliminary surveys showed grazing pressure of the two most dominant grazers, *Evichinus chloroticus* (sea urchin) and *Haliotis iris* (abalone) were relatively low at both the two and 10 metre depth strata along each coast (n = 10 one metre squared quadrats per site). *E*. *chloroticus* densities at the two metre depth strata were 0 and 0.2 ± 0.1 SE per square metre along the modified and forested coasts respectively. *H*. *iris* densities at the same depth were 0.1 ± 0.07 and 0.1 ± 0.05 per square metre along the modified and forested coasts respectively. At the 10 metre depth strata *E*. *chloroticus* densities were 0 and 0.7 ± 0.2 per square metre and *H*. *iris* densities were 0 and 0.03 ± 0.3 per square metre along the modified and forested coasts respectively.

### In situ irradiance

Light data were collected from 14 December 2012 to 7 October 2013. Light intensity and temperature were recorded at the surface, two and 10 metres below mean low water at each site using a data logging sensor (HOBO Pendant Temperature/Light Data Logger 64k, Onset). Using SCUBA, a 25cm aluminium stand was driven into a rock crevice and fixed with underwater epoxy (Concrete Fix, Sika Ltd. Auckland, New Zealand). Each logger was attached using cable ties, with the sensor parallel to the water surface. Surface data loggers were installed in an unshaded location on the shore with the same aspect as the subtidal loggers. Data loggers were programmed to log at 10 minute intervals, each logger was replaced approximately every three months and the data downloaded. This procedure was done to minimise fouling of the logger by algae and invertebrates. Canopy and understory macroalgae were cleared in a two metre diameter around the logger stand when loggers were replaced to avoid direct shading of the logger.

Data logging sensors recorded light intensity in Lux and therefore calibration was required to convert to relevant PAR values. Calibration was achieved through simultaneous recording using HOBO Pendant Temperature/Light Data Loggers and a factory calibrated, cosine corrected, LI-COR underwater quantum sensor (LI-192SA coupled with a LI-250A light metre, LI-COR). For calibration a HOBO data logger, secured parallel to the LI-192SA was programmed to record at one second intervals while the LI-192SA took an average recording over a 15 second period. The corresponding 15 data points from the HOBO logger were averaged for calibration with the LI-192SA. Data from four calibration campaigns, over a range of light intensities, water depths and locations were pooled (S1 Fig). An empirical conversion between Lux and PAR (mol photons m^-2^ s^-1^) was obtained via linear regression of natural log-transformed values [51] using the R statistical software package (v. 3.0.2, R Core Team, 2013). This was then applied to all data. A period of 52 days (27 August 2013 to 18 October 2013) worth of light data from the two metre depth strata at Aramoana was lost due to logger malfunction.

### Macroalgal survey and collection

Macroalgal surveys and collections were conducted in December 2012, April, July and October 2013 at each site. Depth distribution analysis was conducted using SCUBA, three transect lines, each separated by five metres, were laid parallel to the slope of the reef starting at two metres depth running down to 10 metres depth. The presence of all macroalgal species within a one square metre quadrat were recorded at one metre depth intervals between two and 10 metres. The maximum and minimum depth limit and range for each species was calculated from the deepest and shallowest depth recording of that particular species from at least one transect line, at one site, along each coast, over all four sampling periods. Algal collections were conducted by laying a 10 metre transect line perpendicular to the slope of the reef at two and 10 metres depth. Six one square metre quadrats were randomly placed along each transect. All fleshy macroalgae, excluding canopy forming *Macrocystis pyrifera*, were manually removed and placed in a fine mesh bag. Canopy forming *Macrocystis pyrifera* were not included in collections due to their large size and biomass, making collection by divers difficult. However, sub canopy *Macrocystis pyrifera* was collected. Samples were transported directly to the laboratory, classified to the species level, excess water shaken off and then weighed. 15 individuals of each species, over a range of sizes, were weighed wet and then dried in an oven at 60°C until a constant weight was reached. A conversion factor for each species was calculated from the average change in wet to dry weight, this was then applied to all wet biomass data to determine total dry biomass as drying all collected algae was impractical. Seasonal dry biomass was calculated by averaging replicate sites within each coast. Seasonal individual dry biomass was calculated by dividing dry biomass per square metre by the density of individuals per square metre (both pooled by coast) to give an estimate of average individual dry biomass. Seasonal diversity was assessed using the Shannon-Wiener diversity index (*H’*). The Shannon-Wiener diversity index combines species richness and, in this case, relative biomass to give an estimate of diversity, with a value of 0 representing a population of one species. The assumptions of this index are that individuals are sampled from an ‘infinitely large’, random population and that all species from the community are included in the sample [52].

### Statistical analysis

Daily dose of light (mol photons m^-2^ day^-1^), henceforth referred to as daily dose, was calculated by integrating the calibrated 10-minute readings across an entire day. Initial inspection of the raw data showed that sites along the same coast were similar and so data were pooled (hereafter: modified and forested). To visualise differences between coasts, a weighted least-squares regression smoother (loess) with a span (α) of 0.5 (i.e. 50% of the entire data set) and tricubic weighting was applied. Daily integrals of light were used to calculate percent surface irradiance at each depth and a down-welling attenuation coefficient (K_d_) was calculated for each day using the following formula (Kirk 2011):

$${\text{K}}_{\text{d}}\text{=}\frac{\text{In}\left({\text{I}}_{\text{2}}{\text{/I}}_{\text{10}}\right)}{\left(10-\text{ 2}\right)}$$

where I_2_ and I_10_ are the daily doses calculated for two and 10 metres, respectively. The Lambert–Beer equation and calculated K_d_ coefficients were used to provide an estimate of percent surface irradiance throughout the water column. Empirical 90% confidence intervals calculated from lower 5% and upper 95% sample quantiles of daily K_d_ values were used to compare mean estimates from modified and forested coasts.

Seasonal differences in dry biomass, individual weight, density and diversity were tested at each depth strata using a nested analysis of variance (ANOVA), whereby site was nested within coastal region (coast). All factors were treated as fixed. Tukey HSD tests were carried out to test for differences among sites within a coastline and between seasons within a coastline (JMP Pro 10, SAS). Dry biomass, individual dry weight, density and diversity data were log transformed to conform with the assumptions of the parametric tests. All means presented are followed by the value of the standard error of the mean.

## Results

### In situ irradiance

**Fig 2:**
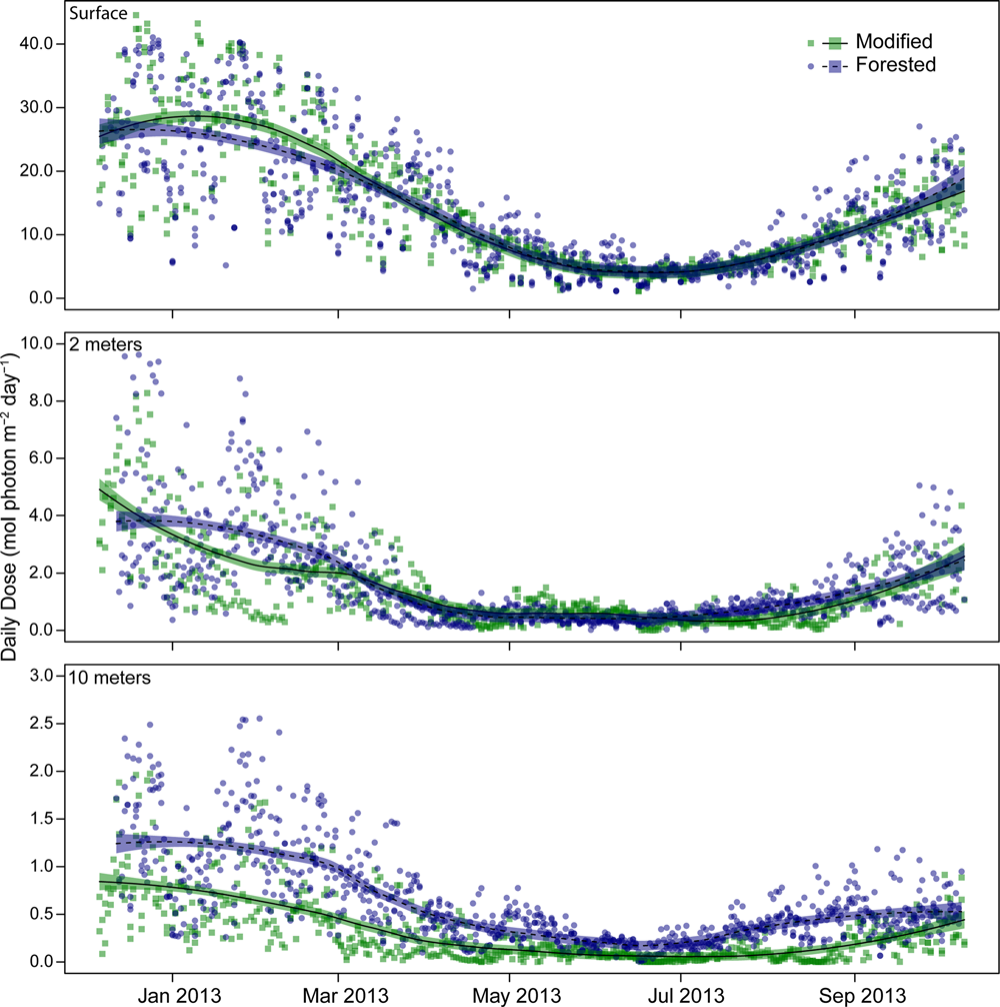
Daily dose mol photons m^−2^ day^−1^ pooled by coast at the surface (top), two metre (middle) and 10 metre (bottom) depths from December 2012 to October 2013. Logging interval for each logger was 10 minutes, deployment of 295 days. Lines represent a weighted least-squares regression smoother (loess, see text for details). Shaded area represents 1.96-times the standard error (approximate 95% C.I.) of the loess smoother. Modified (green, solid line) *n* = 2, forested (blue, dashed line) *n* = 3.

**Fig 3:**
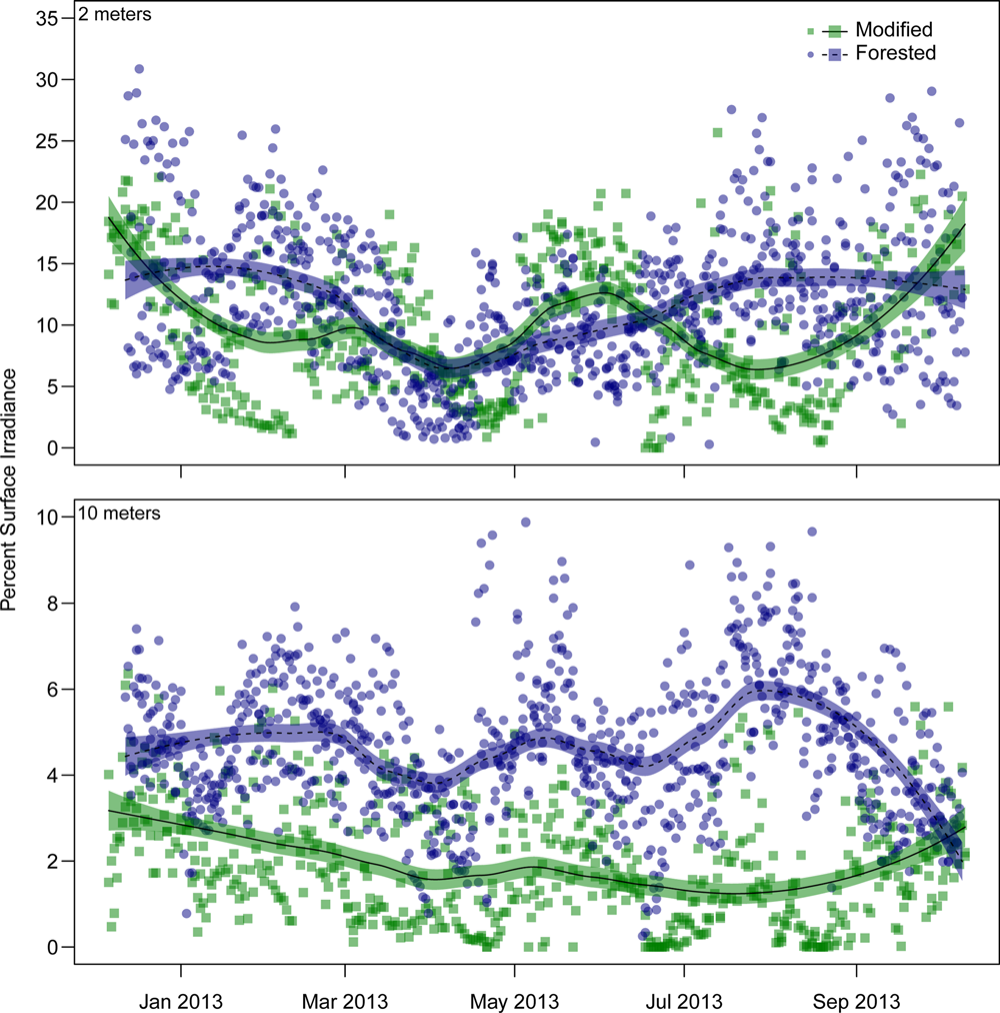
Percentage of surface irradiance pooled by coast at two metre (top) and 10 metre (bottom) depths from December 2012 to October 2013. Lines represent a weighted least-squares regression smoother (loess, see text for details). Shaded area represents 1.96-times the standard error (approximate 95% C.I.) of the loess smoother. Modified (green, solid line) *n* = 2, forested (blue, dashed line) *n* = 3.

**Fig 4:**
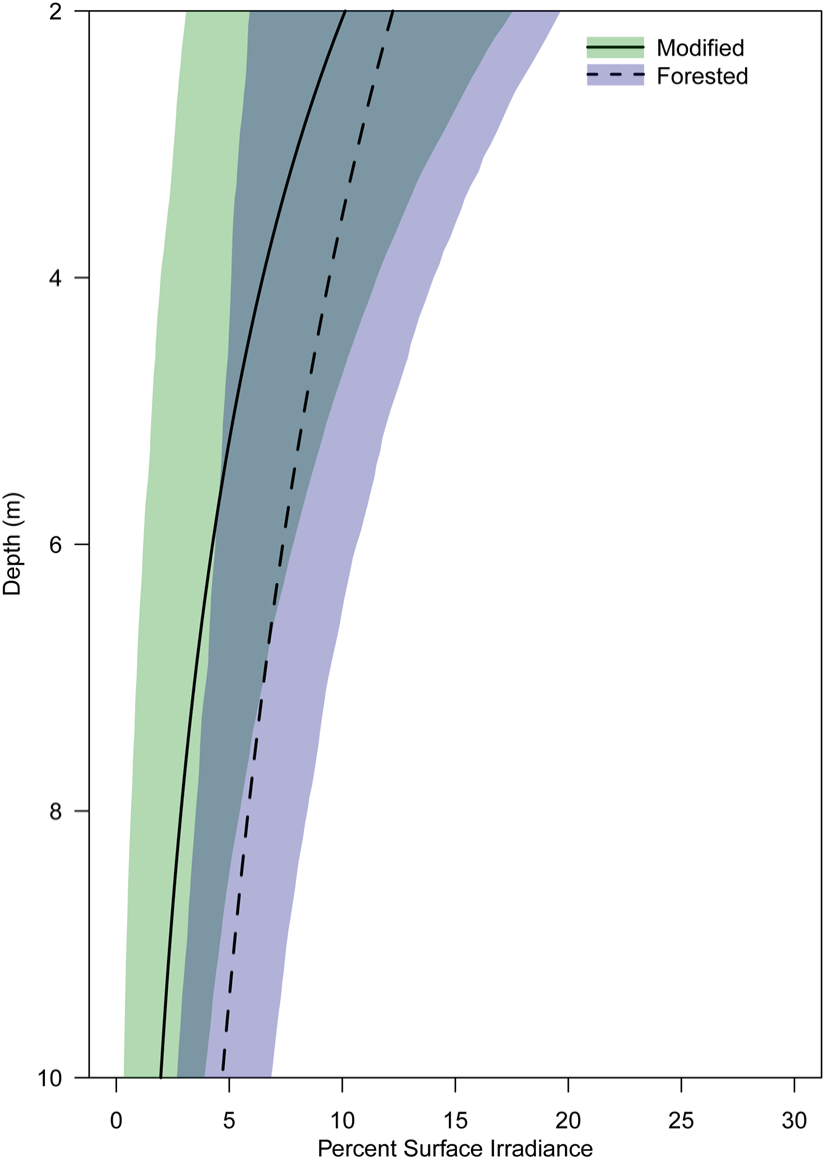
Percentage of surface irradiance, predicted from attenuation coefficients between two and 10 metre depths, pooled by coast between December 2012 to October 2013. Shaded areas represent empirical 90% confidence intervals for each region. Modified (green, solid line) *n* = 2, forested (blue, dashed line) *n* = 3.

A clear difference in average daily dose between modified and forested coasts occurred within the 10 metre depth strata, with values of 0.30 ± 0.01(*n* = 590) and 0.61 ± 0.02 (*n* = 885) mol photons m^-2^ day^-1^, respectively (Fig 2). This resulted in an average quantum dose within the forested coast that was twice that of the modified coast, 179 mol photons m^-2^ and 89 mol photons m^-2^ respectively, for the duration of the study. The difference in quantum dose between coasts is explained by differing attenuation, with more than twice as much of the corresponding surface irradiance reaching the benthos within the forested coast compared to the modified coast (1.93 ± 0.06% (*n* = 590), modified and 4.65 ± 0.06% (*n* = 885), forested) (Figs 3 and 4). Variability in attenuation decreased with depth in both coasts, with a more pronounced difference between coasts at greater depths, shown by the overlapping 90% confidence intervals (Fig 4). The modified coast had a consistently higher rate of attenuation compared to the forested coast at all depths (Fig 4). There was a total of 14 days where light was undetectable within the 10 metre depth strata of the modified coast. The longest period of no light was recorded within the 10 metre depth strata at Karitāne, this period lasted eight days from the 17 to the 24 June 2013. At no point during this study did daily light reach zero within any of the forested sites during daylight hours.

Within the two metre depth strata there was a less pronounced difference in average daily dose between coasts for the duration of the study, with the modified and forested coasts receiving 1.32 ± 0.06 (*n* = 538) mol photons m^-2^ day^-1^ and 1.56 ± 0.06 (*n* = 885) mol photons m^-2^ day^-1^, respectively (Fig 2). This difference resulted in an average quantum dose of 336 mol photons m^-2^ for the modified coast and 396 mol photons m^-2^ for the forested coast over the duration of the study. The percentage of surface irradiance reaching two metres was highly variable between coasts over the duration of this study, with the average values being 9.72 ± 0.24% and 11.7 ± 0.2% for the modified and forested coasts, respectively (Figs 3 and 4). There were four days when light was undetectable within the modified coast during daylight hours, this phenomenon was not seen within the forested coast. The surface light environment of the modified and forested coasts were similar for the duration of this study, the average daily dose being 14.0 ± 0.42 (*n* = 590 days over two sites) and 13.6 ± 0.33 (*n* = 886 days over three sites) mol photons m^-2^ day^-1^, respectively (Fig 2).

### Macroalgal Standing biomass

**Fig 5:**
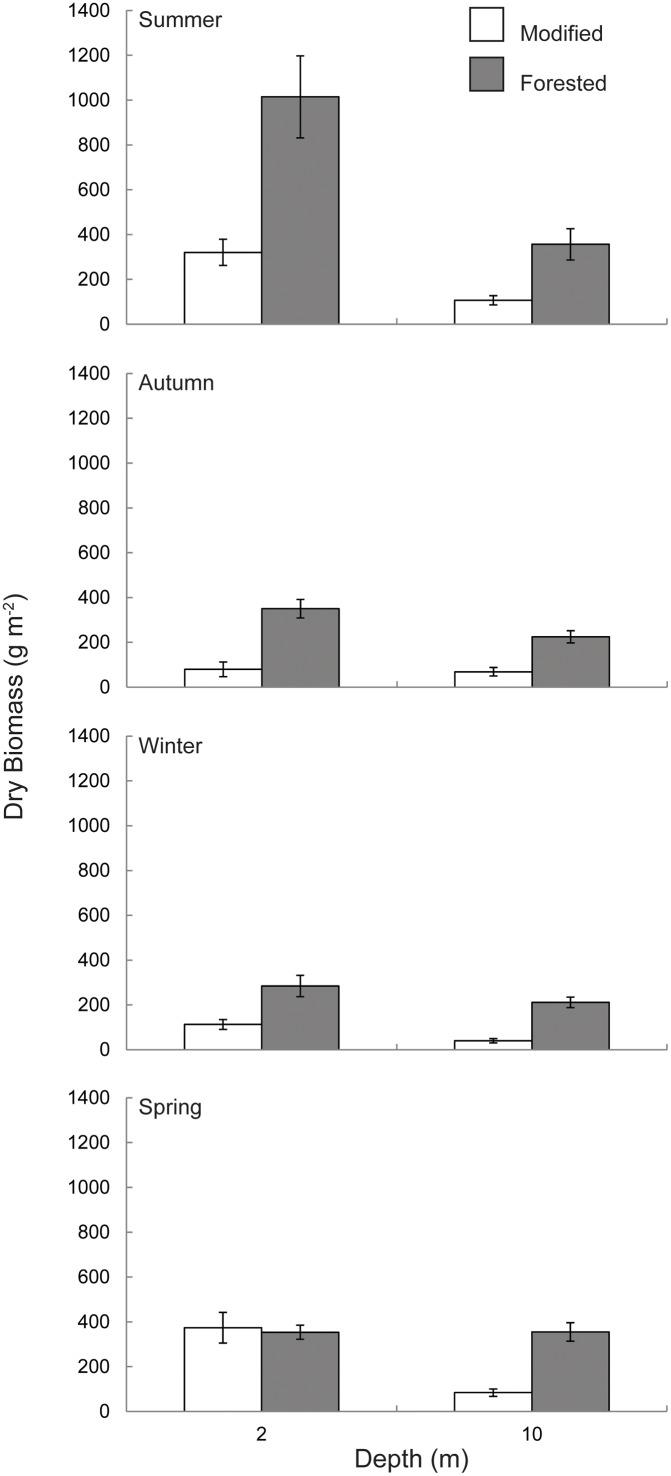
Seasonal macroalgal dry biomass per square metre at (A) two metres and (B) 10 metre depth within the modified coast (white bars) and forested coast (grey bars), New Zealand. Error bars represent mean ± SE for modified *n* = 12, forested *n* = 18.

**Fig 6:**
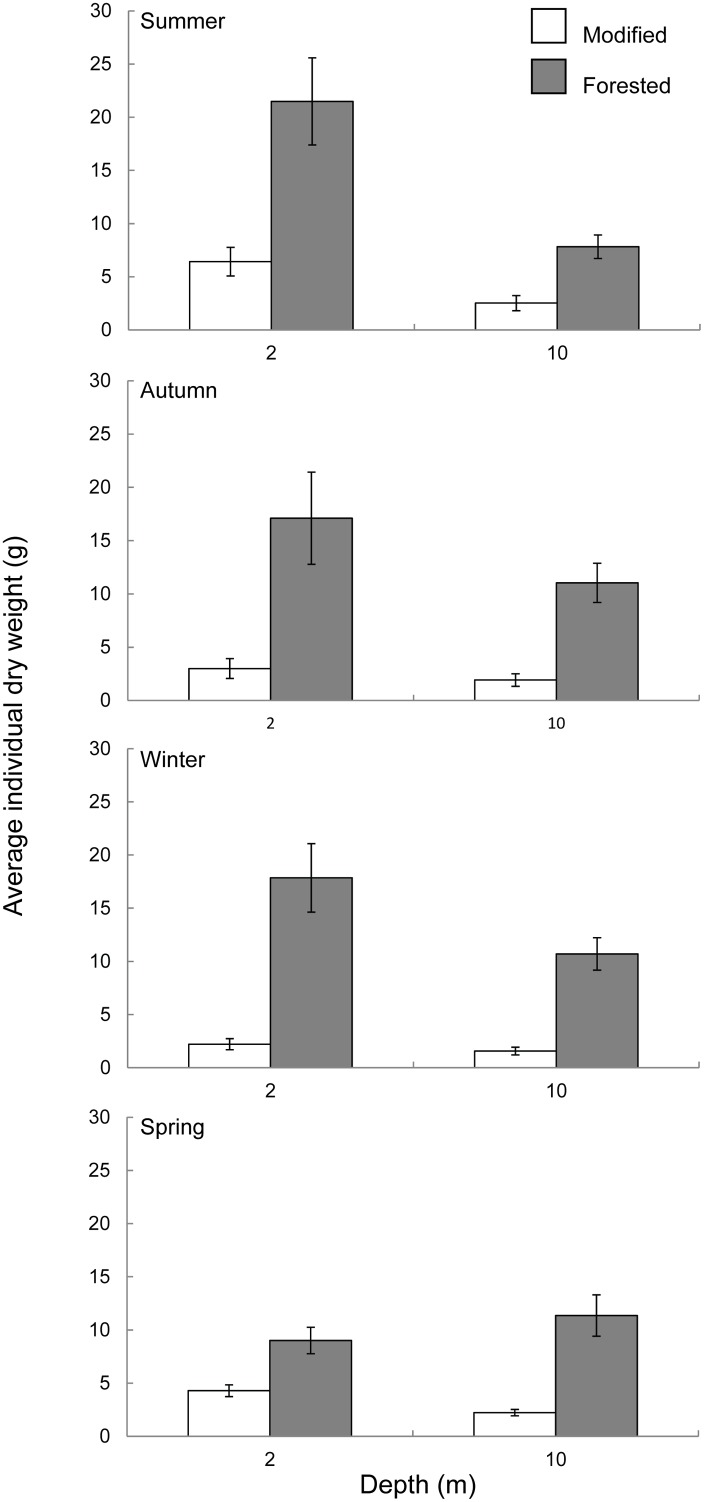
Seasonal individual macroalgal dry weight at (A) two metres and (B) 10 metre depths within the modified coast (white bars) and forested coast (grey bars), New Zealand. Error bars represent mean ± SE for modified *n* = 12, forested *n* = 18.

Within the 10 metre depth strata the forested coast had significantly greater standing biomass compared to the modified coast over all seasons (Fig 5B, Table 1). The same trend was seen within the two metre depth strata during summer, autumn and winter (Fig 5A, Table 1). These differences equated to approximately 2–4 times and 3–5 times greater standing biomass within the forested coast at two and 10 metres respectively. Seasonal standing biomass followed a similar trend to daily dose at both the two and 10 metre depth strata, with peak biomass occurring during summer, declining in autumn and winter then increasing again during spring (Figs 2 and 5). There was no significant difference in standing biomass between sites nested within the forested coast at either two or 10 metres, however within the modified coast Aramoana had significantly higher standing biomass when compared to Karitāne within the 10 metre depth strata (Table 1). Individual macroalgae were on average 3–4 times and 5–6 times heavier within the forested coast at two and 10 metres respectively (Fig 6, Table 1). A significant difference between sites nested within the forested coast was observed with Horseshoe Bay having heavier individuals when compared to Cooper Bay and West Head (Table 1).

**Table 1:** Results of ANOVA on macroalgal community parameters within the two and 10 metre depth strata from the modified and forested coasts.

Community Parameters	Depth (m)	Factor	*F*	p
Dry Biomass	2	Coast	40.92	**<0.0001**
		Site [Coast]	1.46	0.2285
		Season	17.31	**<0.0001**
		Season x Coast	5.38	**0.0017**
Dry Biomass	10	Coast	220.19	**<0.0001**
		Site [Coast]	32.55	**<0.0001**
		Season	12.79	**<0.0001**
		Season x Coast	1.99	0.1196
Individual Dry Biomass	2	Coast	33.08	**<0.0001**
		Site [Coast]	4.72	**0.0039**
		Season	0.87	0.4598
		Season x Coast	3.36	**0.0214**
Individual Dry Biomass	10	Coast	89.75	**<0.0001**
		Site [Coast]	3.07	**0.0311**
		Season	0.16	0.9236
		Season x Coast	3.59	**0.0159**
Density	2	Coast	16.63	**<0.0001**
		Site [Coast]	2.82	**0.0423**
		Season	8.59	**<0.0001**
		Season x Coast	3.82	**0.012**
Density	10	Coast	1.48	0.2269
		Site [Coast]	9.93	**<0.0001**
		Season	7.19	**0.0002**
		Season x Coast	1.12	0.3452
Shannon's Diversity Index	2	Coast	0.38	0.1684
		Site [Coast]	4.62	**0.0044**
		Season	2.46	0.066
		Season x Coast	0.67	0.5734
Shannon's Diversity Index	10	Coast	0.22	0.6424
		Site [Coast]	20.12	**<0.0001**
		Season	3.03	**0.0326**
		Season x Coast	4.02	**0.0093**

Factors were Coast (df = 1), Site nested within Coast (df = 3), Season (df = 3) and Season crossed with Coast (df = 3). Significant interactions are **Bold**.

### Macroalgal community structure

**Fig 7:**
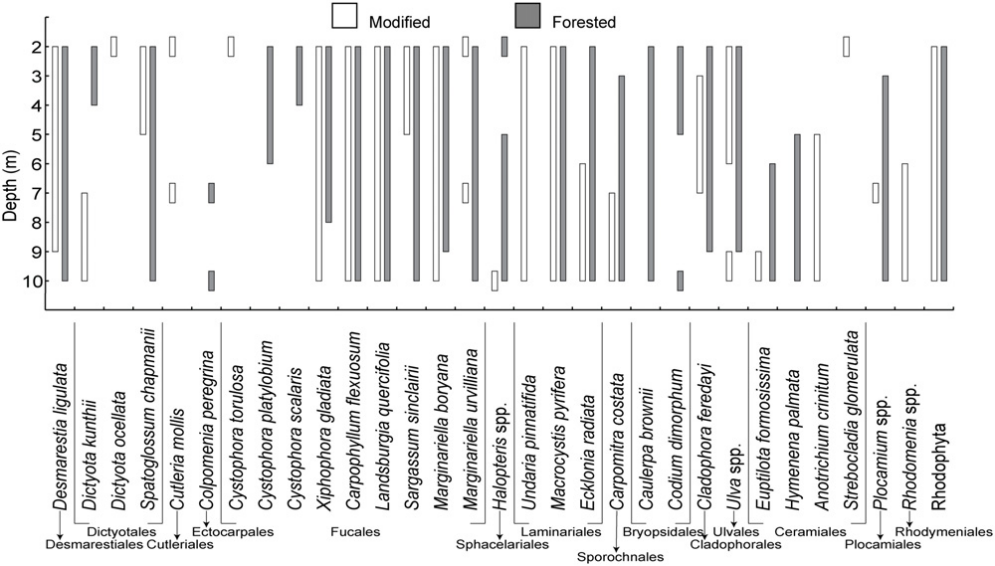
Maximum and minimum depth distribution of dominant macroalgal species over all four seasons within the modified coast (white bars) and forested coast (grey bars), New Zealand. Modified *n* = 6, forested *n* = 9.

The depth range of species shared by both coasts was greater within the forested coast with the average range of a species spanning 6.9 ± 1.63 metres while within the modified coast the average range was 4.7 ± 0.65 metres (Fig 7). The depth limit of the shared species was on average 0.9 ± 1.08 metres deeper within the forested coast compared to the modified coast. Of the dominant species recorded during depth distribution analysis the two coasts shared 18 species, seven species within the modified and six within the forested coast were not shared.

A total of 56 macroalgal species were recorded within the two and 10 metre depth strata from the modified (38 species) and forested (45 species) coasts during the sampling period. The only significant difference in macroalgal density between coasts was observed during winter at the two metre depth strata, where the modified coast had a higher density of individuals per square metre compared to the forested coast (Table 1). A significant difference between sites nested within coast was observed, with Aramoana having a greater density of species than Karitāne at the 10 metre depth strata (Table 1). For the majority of the of the sampling period there was no difference in the Shannon-Wiener Diversity index between coasts, the exception being within the 10 metre depth strata during spring when the forested region had a significantly higher *H’* value compared to the modified coast (Table 1). A significant difference between sites nested within coast was observed with Karitāne having a higher *H’* value than Aramoana at both two and 10 metre depth strata (Table 1).

## Discussion

Attenuation of light through the water column was consistently lower on the forested coast resulting in an average daily dose and total quantum dose (total amount of light received over the entire study) approximately twice that of the modified coast at 10 metres depth. A less pronounced difference in light availability between the forested and modified coast was observed at the two metre depth strata, however the forested coast still received 20% more light than the modified coast. The variability of light reaching two metres was greater than at 10 metres within both coasts, this was in part attributed to light scattering caused by wave action and the increased dose of light received at two metres [2,22,23]. The major difference, and by far the most important in terms of potential productivity and habitat provision was the contrast in biomass per square metre of substrate at relatively shallow depths between the modified and forested coasts [19,53]. This result was purely due to the size of individual macroalgae rather than differences in density. Communities within the forested coast were made up of individuals that were on average three times the biomass of individuals from the modified coast. Additionally, dominant species found on both coasts were distributed over a greater depth range and grew deeper within the forested coast compared to modified coast. It must be noted however that many species true depth limits were not determined as the reef extent was to only 10 metres and, particularly on the forested coast, species were highly abundant and individuals did not appear stunted at this depth, indicating they could likely grow deeper. Macroalgae on the forested coast provide a more extensive three dimensional habitat, over a greater depth range and potentially a greater depth limit, inferring a greater contribution of energy to the coastal food web at a community level when compared to the modified coast [16,54].

Biomass tracked daily dose with a peak during summer and a minimum during autumn and winter, a trend consistent with other studies [19,52,54]. Despite the proportional difference in light dose between forested and modified coasts being greater at 10 metres compared to two metres the absolute differences in the availability of light at these depths was relatively similar between coasts over the sampling period (60 mol photons m^-2^ at two metres and 90 mol photons m^-2^ at 10 metres). Differences in biomass observed at two metres are therefore consistent with differences in the availability of light (dose) between the forested and modified coasts.

Of interest is the fact that during the spring sampling period macroalgal biomass at two metres was not significantly different between the modified and forested coasts. There was a significant difference in biomass among the modified sites during this period, with Aramoana having almost twice the biomass of Karitāne at two metres. At the Aramoana site, *Undaria pinnatifida* (Harvey) Suringar, an invasive Asian kelp, made up 77% of the total biomass, this species was not present within any other site. The high variability (at a coastline scale) is indicative of lowered stability and a substantial change in community structure, highlighted by the successful invasion of *U*. *pinnatifida* [54–57]. It is possible that decreased light availability may have aided in the successful invasion of *Undaria pinnatifida* by excluding native species with less efficient light harvesting abilities and lower maximal photosynthetic rates [58,59].

Analysis of diversity through the use of the Shannon-Wiener diversity index showed that the only difference between coasts occurred during spring within the 10 metre depth strata. This means that diversity and the evenness of spread, at a species level was not significantly different between coasts during summer, autumn and winter. However a subsequent study comparing the compositional structure at both the species and functional group level within one site from the modified and one from the forested coast found that these sites varied in their respective species and functional contribution to total standing biomass. Through the use of multivariate statistics it was shown that large leathery species provided a greater contribution to total standing biomass within the highlight (forested) compared to the low light (modified) site (Desmond et al. in review). These findings suggest that common and relatively easily obtained metrics such as species diversity and density, used to describe kelp forest communities [39,60,61], may not be sufficient when investigating the effects of light limitation on ecosystem functioning and community composition. Of more importance is high resolution data regarding species specific biomass and depth distribution [19,53,62], as these provide a more accurate estimate that can be used to determine the potential productivity of a particular ecosystem.

This study provides the first community-level comparison of macroalgal depth distribution, composition and biomass in relation to the specific underwater light environment of kelp dominated rocky reef systems that differ in light availability. The relationship between long term availability of light (dose) and biomass, even at shallow depths is a very interesting result and is reflective of the strong absorption and scattering of light that occurs in the first metre of the water column. These key differences in light dose are unlikely to be detected with typical short term and one off measurements and understanding the mechanisms behind and the extent of light attenuation in shallow water (< 2 metres) warrants further attention. This study has demonstrated the potential these locations hold for teasing apart causal mechanisms of light limitation e.g. potential effects of catchment land use, through a baseline versus modified study approach. Unfortunately the lack of unmodified coastal catchments makes replication at the coastline level problematic within the study area. Without spatially interspersed modified and unmodified catchments it is a challenge to understand the effects of potential spatially confounding variables such as recruitment dynamics and it must be noted that this limitation is experienced globally and is ever increasing [10,40]. This limitation should not restrict such studies as it is vital we understand how unmodified systems function in order to better understand the effect we have on systems we have modified. Further work is now required to quantify the potential influence of other factors such as sedimentation and wave exposure which play an important role in recruitment and productivity of kelp ecosystems [1,9].

In times of such unprecedented coastal development and population growth it has never been more important to understand the fundamental factors controlling marine productivity and ecosystem functioning and how anthropogenic forces influence such factors. It is critical that we continue to grow and develop while preserving and enhancing the productivity of coastal oceans which we rely so heavily upon. Given that this information has such potential to inform the most basic of predictions regarding coastal primary productivity, it is surprising how little is known about the temporal and spatial variability of light within these systems.

## Supporting Information

S1 Fig Calibration of natural log-transformed data from HOBO and LI-COR light data loggers over four campaigns via linear regression. (TIF)
