# Supplementary figures and images for: Light Limitation within Southern New Zealand Kelp Forest Communities
Source: PLoS One. 2015 Apr 22;10(4):e0123676. doi: 10.1371/journal.pone.0123676 (PMC4406696; doi:10.1371/journal.pone.0123676)

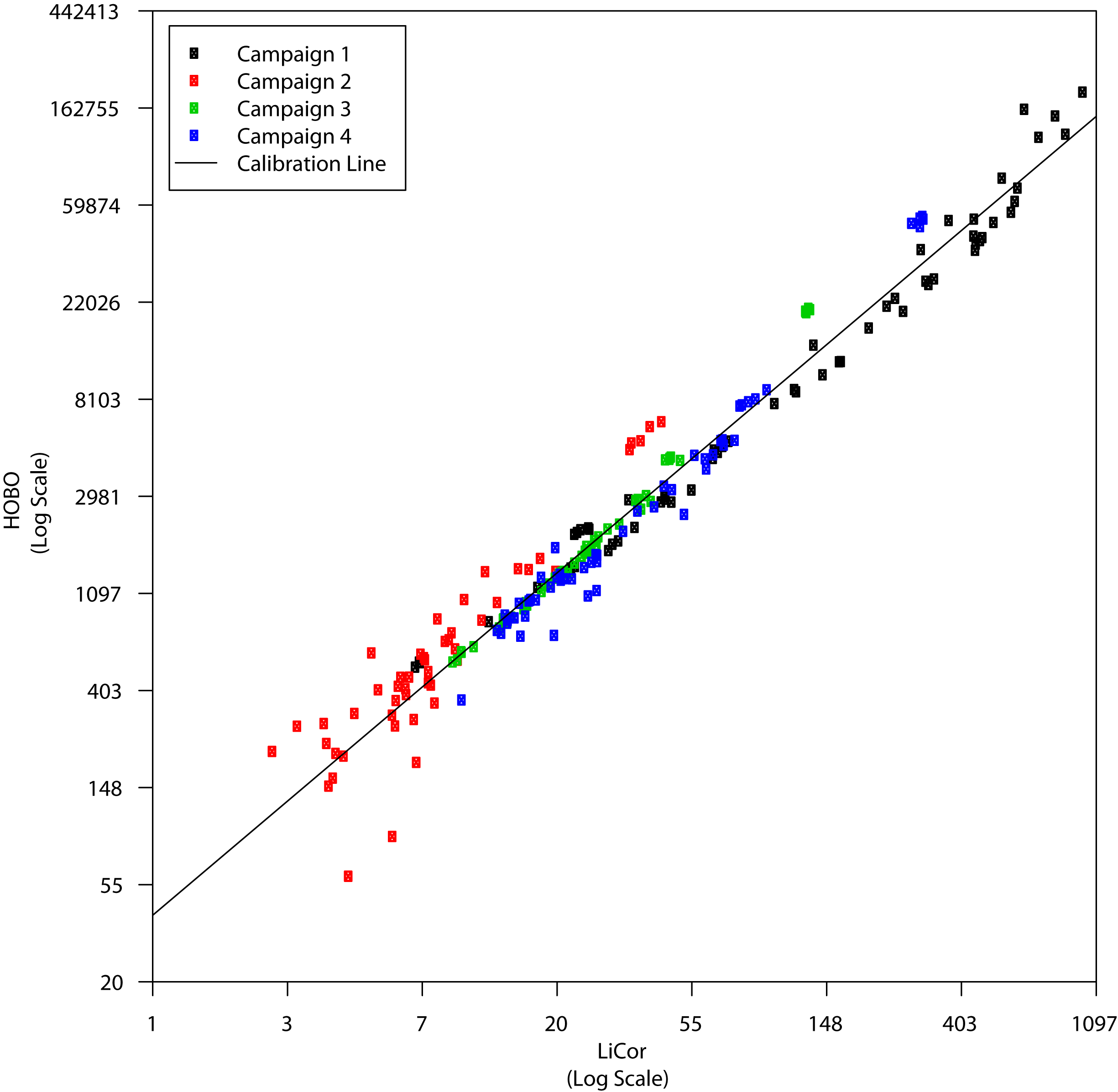

Supplement: S1 Fig — (TIF) [file pone.0123676.s001.tif]
